# Host Suitability and Parasitic Activity of Local Groups of *Habrobracon hebetor* (Say, 1836) (Hymenoptera: Braconidae) as a Factor in the Management of Lepidopteran Pest Populations in Corn Agroecosystems

**DOI:** 10.3390/insects17101012

**Published:** 2026-09-30

**Authors:** Maria Vladimirovna Petrishcheva, Vladimir Yakovlevich Ismailov, Irina Sergeevna Agasyeva

**Affiliations:** Federal State Budgetary Scientific Institution «Federal Research Center of Biological Plant Protection», 350039 Krasnodar, Russia; vlyaism@yandex.ru (V.Y.I.); agasieva5@yandex.ru (I.S.A.)

**Keywords:** *Habrobracon hebetor*, biological protection of plants, ectoparasitoid, host suitability, parasitic activity, corn, *Ostrinia nubilalis*, *Helicoverpa armigera*

## Abstract

*Habrobracon hebetor* (Say, 1836) (Hymenoptera: Braconidae) is one of the most significant parasitoids of Lepidoptera in agroecosystems, whose role in the biological control and management of pest populations requires a detailed study of the ecological and biological characteristics of local populations. The paper presents the results of a study of the biological features of natural ectoparasitoid populations captured on corn crops in two districts of the Krasnodar Territory (Russia). The host range and parasitic activity of isolated populations against Lepidoptera caterpillars from the families Noctuidae, Pyralidae, and Tortricidae have been evaluated under laboratory conditions. It has been established that there are pronounced differences in host suitability within *H. hebetor*: one population (pyralid-leafroller) effectively infects the European corn borer, pulse pod borer moth, and leafroller moths, but does not infect the cotton bollworm; the other (owlet-pyralid) actively parasitizes the cotton bollworm, European corn borer, and pulse pod borer moth. High paralyzing activity (up to 100%) and parasitization (up to 91%) have been shown in relation to caterpillars of middle and older ages. In comparative field observations under release conditions, at a release rate of 1400 individuals/ha, damage by the European corn borer was observed in 16% of corn plants in the treated plot compared with 40% in the control plot. Damage by the cotton bollworm was observed in 13% of corn ears in the treated plot compared with 65.0% in the control plot. The data obtained indicate the potential of using local populations of *H. hebetor* as effective bioagents for biological control of economically significant Lepidoptera pests in agrocenoses.

## Simple Summary

*Habrobracon hebetor* is a key parasitoid of Lepidoptera, and its biocontrol potential requires understanding local population traits. We studied natural populations from maize fields in two districts of Krasnodar Krai, Russia. Under laboratory conditions, we assessed host range and parasitic activity against Noctuidae, Pyralidae, and Tortricidae larvae. Clear intraspecific differences in host suitability emerged: one population (pyralid-tortricid type) parasitized the European corn borer (*Ostrinia nubilalis*), pulse pod borer moth (*Etiella zinckenella*), and tortricids, but not the cotton bollworm (*Helicoverpa armigera*); the other (noctuid-pyralid type) attacked the cotton bollworm, corn borer, and pulse pod borer moth. Both showed high paralyzing (up to 100%) and parasitization (up to 91%) rates on medium and late instars. In comparative field observations under release conditions, corn borer damage was observed in 16% of plants in the treated plot versus 40% in the control plot, and cotton bollworm damage was observed in 13% of ears versus 65% in the control plot. Our results highlight the promise of native *H. hebetor* populations as potential bioagents against key lepidopteran pests in crop systems.

## 1. Introduction

Among the natural enemies of lepidopteran pests, *Habrobracon hebetor* (Say, 1836) (Hymenoptera: Braconidae) stands out as a particularly effective larval parasitoid. It is widely employed in biological control programs targeting noctuids (Noctuidae) and pyralids (Pyralidae) [1,2]. The species has a long history of mass rearing and application against various lepidopteran pests affecting both field crops and stored products such as grain and tobacco [3,4,5,6]. When released at low densities (1000–3000 individuals per hectare), artificially reared *H. hebetor* can suppress European corn borer, cotton bollworm, and pulse pod borer moth populations by 70–90% [7,8].

Previous studies have shown that different populations of *H. hebetor* have distinct environmental optima, including temperature, humidity, and photoperiod [9,10,11]. For instance, the Moldovan population develops best at 25 °C, 60% relative humidity, and a 20 h photoperiod. In contrast, populations from Tajikistan and Uzbekistan require 27 °C, 60% relative humidity, and a 20 h photoperiod, under which their numbers increase 9.1- and 15.2-fold, respectively [12]. The Stavropol population reaches peak activity at 25–30 °C [13]. Furthermore, the lifetime fecundity of *H. hebetor* is affected by the host species [14,15,16,17].

Notably, *H. hebetor*, in contrast to Trichogramma species, exhibits rapid adaptation to rearing and field conditions [4]. These two entomophages can act synergistically, and their combined releases in cotton agroecosystems have been shown to delay crop infestation by 12–20 days [18]. In Stavropol Krai, releases of Habrobracon resulted in parasitism of 64–87% of noctuid larvae and 68–91% of pulse pod borer moth (*Etiella zinckenella*) larvae [19,20]. Nevertheless, multiple studies have reported considerable variation in the host range of *H. hebetor* under both laboratory and field conditions [21,22,23,24]. Moreover, Heimpel et al. [25] showed that populations initially differing in host-related traits can be reproductively isolated and genetically differentiated; on this basis, the Barbados strain was described as a separate species. This means that differences in host use alone do not necessarily indicate microevolutionary differentiation within *H. hebetor*, and that the studied groups may represent genetically differentiated lines or be part of a species complex. Changes in the biological parameters of this species, such as host suitability, station distribution, and migratory abilities, often complicate its practical use in biological plant protection. In this regard, the study of the biological features of *H. hebetor* will allow us to assess the prospects for its further use in the biological control of the abundance of several Lepidoptera pests.

Researchers from Thailand and Japan describe two genetically different strains of *H. hebetor*, isolated spatially and differing in trophic specialization [26]. This, along with the data of Heimpel et al. [25], points to the possible existence of a species complex within *H. hebetor*. In our study, we examine two groups of *H. hebetor* from different geographical locations in the same region. Thus, despite differences in host suitability and some other biological features, these groups are not spatially isolated. Therefore, to separate the biological agent *H. hebetor* according to its economically useful characteristics, we conditionally divide its intraspecific groups from different points of the Krasnodar Territory (Russia) into owlet moth, pyralid, leafroller, and other groups. At the same time, we deliberately use the neutral term “group” rather than “race” or “species” until data on genetic and reproductive isolation are obtained.

The aim of the study was to assess host suitability and parasitic activity of two local groups of *H. hebetor* collected from corn fields, and their comparative field performance under release conditions against O. nubilalis and H. armigera, in order to substantiate a differentiated approach to managing Lepidoptera pest populations in corn agroecosystems.

## 2. Materials and Methods

The collection of entomophages (*H. hebetor*) and their hosts was carried out in the western part of the Temryuksky district of the Krasnodar Territory (45°26′ north, 37°11′ east), as well as in the central zone of the Krasnodar Territory (45°02′ north, 38°59′ east). For this purpose, cassettes were hung on corn crops (Figure 1) with caterpillars of the European corn borer (*Ostrinia nubilalis* Hubn.) and cotton bollworm (*Helicoverpa armigera* Hubn.) collected on damaged corn plants. Every 7–10 days, the cassettes were collected and delivered to the laboratory, and the collected material was left in containers under supervision. New cassettes were hung instead of the collected ones.

The starting populations of *H. hebetor* captured from nature across various geographical locations of the Krasnodar Territory were isolated as stock cultures, for which mass-breeding methods were developed. The caterpillars of the mill moth (*Ephestia kuehniella*) and the greater wax moth (*Galleria mellonella*) were selected as host insects for laboratory cultivation of the stock populations of the *H. hebetor*.

Initial cultures of *H. hebetor* were established from adults that emerged from parasitized host larvae collected in the field using cassettes with *O. nubilalis* and *H. armigera* larvae. The identification of the captured species was confirmed using the identification keys [27,28].

In total, from each geographical point we obtained: Temryuk district (western zone): 256 founder individuals that emerged from parasitized larvae collected from 15 cassettes; central zone of Krasnodar Krai: 288 founder individuals from 15 cassettes.

Individuals from different geographical points were not mixed. Each colony was established and subsequently maintained separately, which excludes cross-mixing of genetic material between the Temryuk district and the central zone of the krai.

Within each point, all adults that emerged from different cassettes and possibly from different host species were combined into a single laboratory colony. Separate maternal lines were not maintained. This could have led to mixing of genetically distinct lines within a single point, and a founder effect cannot be fully excluded. This limitation should be taken into account when interpreting the obtained results.

Both colonies of *H. hebetor* were reared under identical conditions: temperature 26–28 °C, relative humidity 70%, photoperiod 16 h, using a Binder KMF-240 climatic chamber (BINDER GmbH, Tuttlingen, Germany). Larvae of *E. kuehniella* and *G. mellonella* were used for rearing. The two host species were not alternated or used together: the colony from the Temryuk district was maintained on *E. kuehniella*, and the colony from the central zone of Krasnodar Krai on *G. mellonella*. From the moment of field collection to the start of the experiments, 2 generations developed, meaning that the colonies were only briefly reared in the laboratory to obtain a sufficient number of individuals.

To assess the host range and biological efficacy of different geographical populations of *H. hebetor* in agroecosystems, larvae of several Lepidoptera species from the families Noctuidae, Pyralidae, and Tortricidae were collected and offered to *H. hebetor* for infection under laboratory conditions. The experiments were conducted using a no-choice test design: each parasitoid was offered a single host species in isolation. Host suitability was judged indirectly by the formation of cocoons and the emergence of adult offspring. On those hosts that were paralyzed but on which no offspring developed, oviposition was not observed; thus, the absence of offspring in these cases was due to the female’s refusal to oviposit rather than to the death of laid eggs or larvae. Based on the experimental results, each group of *H. hebetor* differing in host suitability was assigned a conventional group designation.

Of particular interest in our study was a group of parasitoids capable of effectively infecting cotton bollworm caterpillars. Therefore, for this group, we also evaluated the most appropriate age of cotton bollworm caterpillars for parasitoid infection. In laboratory conditions, *H. armigera* caterpillars of various ages (young, middle-aged, and old) were offered to the *H. hebetor*. The effectiveness of infection was judged by the proportion of paralyzed and parasitized caterpillars, as well as by the number of cocoons formed and the imago of the *H. hebetor* that flew out.

Based on the results of the host suitability study, each group of *H. hebetor* was tested under field conditions on isolated corn plots. The entomophage was released at a rate of approximately 1400 individuals/ha.

To test *H. hebetor* against *O. nubilalis*, one treated plot of 4 ha and one spatially separated control plot of the same area were used; therefore, the plot was the experimental unit, and no independent plot-level replication was available. The first release of *H. hebetor* was carried out at the beginning of tassel emergence, and the second release after 10–12 days. Damage levels were compared between the treated and control plots by counting larvae on 25 stems in each of three sampling units within each plot: stems were cut to soil level, split lengthwise to the base, and the number of viable European corn borer larvae in the stems was counted [29]. No protective measures were applied on the control plot (no *H. hebetor* release, no insecticides).

In the experiments to study the field performance of *H. hebetor* under release conditions against *H. armigera*, one treated and one spatially separated control plot of 3 ha each were established; the plot was the experimental unit, and no independent plot-level replication was available. The entomophage was released twice: the first at the beginning of the appearance of first-generation larvae (end of corn flowering—beginning of grain formation), and the second after 5–7 days, during mass hatching of larvae. To activate and reproduce the ectoparasitoid under natural conditions, cassettes with *H. armigera* larvae were hung on the treated plot; parasitic activity of *H. hebetor* under release conditions was determined from records of larval infestation in the cassettes. Damage to corn ears was compared between the treated and control plots; counts were performed on 25 corn ears in each of three sampling units within each plot. On the control plot, no cassettes were hung, and no protective measures against the cotton bollworm were applied (no *H. hebetor* release, no insecticides).

Statistical analysis was performed using Statistica 13.2 and Microsoft Excel 2016 (Microsoft Corporation, Redmond, WA, USA).

## 3. Results

### 3.1. A Study of the Trophic Specialization of H. hebetor

An assessment of various geographical populations of the *H. hebetor* in relation to several species of Lepidoptera of the families Noctuidae, Pyralidae, and Tortricidae allowed us to identify two groups of *H. hebetor* that differ in food specialization: pyralid-leafroller and owlet/pyralid. Thus, the population captured in the Temryuksky district turned out to be pyralid-leafroller. It was found that the pyralid-leafroller population of *H. hebetor* introduced into laboratory culture effectively parasitized the caterpillars of green oak tortrix (*Tortrix viridana* L.), European corn borer (*O. nubilalis*), pulse pod borer moth (*E. zinckenella*), plum fruit moth (*Grapholitha funebrana* Tr.), and codling moth (*Cydia pomonella* L.) (Figure 2).

The proportion of caterpillars parasitized by *H. hebetor* during the infection of the European corn borer was 91.3%, and the number of parasitoids that flew out was 85.6%. When the pulse pod borer moth was infected with *H. hebetor*, the number of caterpillars from which the parasitoid flew out was 85.4%. Positive results were obtained when infecting caterpillars of the codling moth and plum fruit moth, the death rate of which was 67% and 84%, respectively. The olive-shaded bird-dropping moth (*Ponometia candefacta* Hubn.) was paralyzed by *H. hebetor*, but the parasitoid did not develop on it. The test population of the *H. hebetor* also did not develop on the cotton bollworm.

The population captured in the central zone of the Krasnodar Territory exhibited high paralyzing activity against all Lepidoptera pest species included in the experiment (Figure 3).

However, parasitization was noted only on cotton bollworm and pyralid moths (European corn borer, pulse pod borer moth) (Figure 4).

The proportion of paralyzed caterpillars was 100% for *C. pomonella*, *O. nubilalis*, and *H. armigera*, and 97% for *G. funebrana*. However, parasitization (infection with subsequent development of offspring) was not observed in all species. The largest proportion of parasitized caterpillars was found on *O. nubilalis*, at 91%, on *H. armigera*, at 87%, and on *E. zinckenella*, at 55.6%. At the same time, no parasitoid development occurred on the caterpillars of *C. pomonella* and *G. funebrana* (the proportion of parasitized specimens was 0%), despite their complete or almost complete paralysis.

Thus, the tested population of *H. hebetor* effectively realizes its reproductive potential primarily on caterpillars of owlet moths and pyralid moths, and can be classified as an owlet–pyralid group. The data obtained confirm the presence of intraspecific differences in host suitability and indicate the need for preliminary testing of each population before its practical application in biological plant protection.

### 3.2. Assessment of Parasitic Activity of H. hebetor Depending on the Age Structure of Cotton Bollworm Caterpillars

The timing of the release of entomophages and the developmental phases of the target insect are of paramount importance, especially for maximizing the effect of the use of parasitic Hymenoptera.

In laboratory experiments, it was found that *H. hebetor* effectively paralyzed cotton bollworm caterpillars of middle and older ages, with parasitoid flight rates of 54.5% and 78.1%, respectively (Table 1).

Young caterpillars were not infected by *H. hebetor*: the proportion of paralyzed and parasitized caterpillars was 0%.

Thus, a population of *H. hebetor* captured in nature and bred in the laboratory, which we classified as an owlet-pyralid population, effectively infected *H. armigera* caterpillars of middle and older age and met all the requirements for use as a bioagent to regulate the number of cotton bollworm.

### 3.3. Evaluation of the Field Effectiveness of Various Populations of H. hebetor for the Protection of Corn from H. armigera and O. nubilalis

In comparative field observations with the pyralid–leafroller group of *H. hebetor* released against the European corn borer, it was found that the ectoparasitoid could penetrate corn ears and stalks, paralyze and infect the caterpillars within them. *H. hebetor* quickly dispersed, fed on flower nectar, and concentrated in foci of increased pest population density. The released parasitoid actively reproduced and, during the development period of one generation of the European corn borer, developed in 2 generations. Damage to corn plants by *O. nubilalis* was observed in 16% of plants in the treated plot compared with 40% in the control plot.

In comparative field observations with the owlet–pyralid group of *H. hebetor* released on corn against the cotton bollworm, the parasitic activity of *H. hebetor* reached a maximum about two weeks after release and remained at a high level. The damage to corn ears by *H. armigera* was observed in 65.0% of ears in the control plot and 13.0% in the treated plot. Under release conditions, the proportion of parasitized cotton bollworm larvae was 87.0%, and the proportion of emerged adults was 88.3%. Thus, on average, of the 69.0 caterpillars hung in cassettes, two weeks after the release of *H. hebetor*, all were paralyzed, with the proportion of parasitized being 87.0% (Table 2).

As can be seen from the data presented in Table 2, the owlet–pyralid group of *H. hebetor* was associated with infection and development of cotton bollworm caterpillars under the field conditions of the corn agrocenosis: 234.3 cocoons formed on 69.0 caterpillars, from which 206.9 *H. hebetor* specimens flew out.

## 4. Discussion

In our work, for the first time, two groups were identified in the Krasnodar Territory that differed in their host spectra: the pyralid-leafroller population (Temryuksky district) and the owlet-pyralid population (central zone of the region).

The differences in host suitability between the two groups of *H. hebetor* that we obtained are consistent with numerous studies by other authors [14,15,16,17,18,19,20,21,22,23,24,26] and with the data of Heimpel et al. [25], who showed that populations differing in host-related traits can be reproductively isolated and genetically differentiated. However, unlike the aforementioned study, we do not yet have data on reproductive isolation or molecular-genetic differentiation between the studied groups. Therefore, we cannot exclude that the observed differences reflect either microevolutionary processes within a single species or the presence of a species complex. This issue requires further study using molecular-genetic methods and tests of reproductive compatibility.

Special attention should be paid to the fact that both groups effectively paralyze a wide range of hosts. Still, reproductive potential (parasitization and offspring development) is not realized in all species. This indicates that paralysis and parasitization proper are two different biological processes, and the former does not always lead to the latter. This phenomenon requires further study at the physiological and biochemical levels (possibly involving differences in host hemolymph composition or in immune system responses).

Our laboratory experiments have shown that the owlet-pyralid population of *H. hebetor* does not infect caterpillars at younger ages (0% paralysis and parasitization). In contrast, at middle and older ages, the effectiveness ranges from 54.5% to 78.1% in adult emergence. Similar patterns have been observed in other populations of *H. hebetor*: females prefer older hosts, and young hosts become infected much less frequently [30,31]. Furthermore, *H. hebetor* regulates egg-laying size (the number of eggs laid per host) in response to the host’s size and quality [32,33]. This can be explained by the limited size of the younger caterpillars; probably, such small caterpillars do not provide sufficient nutrient substrate for the development of parasitoid larvae.

The data obtained are of great practical importance: to maximize the effectiveness of biological control, releases of *H. hebetor* should be synchronized with periods when middle-aged and older caterpillars predominate in the cotton bollworm population. In the Krasnodar Territory, this corresponds to the end of corn flowering and the beginning of grain formation (the first decade of July for the first generation and the end of July–August for the second generation).

In the comparative field observations under release conditions, at a release rate of 1400 individuals/ha, damage to corn plants by the European corn borer was observed in 16% of plants in the treated plot compared with 40% in the control plot, where no protective measures were applied. Damage to corn ears by *H. armigera* was observed in 65% of ears in the control and 13% in the treated plot. Because these field comparisons used one treated plot and one control plot, and the three assessments per plot were sampling units rather than independent plot-level replicates, these observations provide preliminary field evidence and should not be interpreted as experimentally demonstrated treatment efficacy. They indicate that damage levels differed between the treated and control plots under the conditions of this study, but causal attribution of the observed differences to *H. hebetor* release is limited by the lack of independent replication at the plot level. Against the cotton bollworm under release conditions, the proportion of parasitized larvae was 87.0%, and the proportion of emerged adults was 88.3%. These values are comparable to or exceed published data. Thus, according to Kovalenkov et al. [19] and Kil et al. [7] for Stavropol Krai, *H. hebetor* infected 64–87% of owlet moth larvae and 68–91% of pulse pod borer moth larvae. Our values fall within the upper range of these figures, which indicates a high biocontrol potential of the local Krasnodar groups of *H. hebetor*. It should be emphasized, however, that no cassettes with larvae were hung on the control plot against the cotton bollworm, so it was not possible to assess the natural parasitism rate in the absence of releases. In this regard, the obtained parasitism values should be interpreted specifically as results obtained under release conditions, and not as biological efficacy in the strict sense.

It is important to note that in our field experiments to study the effectiveness of the owlet-pyralid population against the cotton bollworm, we used not just a double release but a technique with supporting cassettes (with host caterpillars) that served as reserves for the parasitoid’s reproduction. This made it possible not only to evaluate the immediate effectiveness but also to demonstrate the ability of *H. hebetor* to maintain a population within an agrocenosis. The data obtained are consistent with the conclusions of Patrashko [18] that *H. hebetor* can quickly adapt and concentrate in pest foci.

The method we have developed for trapping natural populations using cassettes containing host caterpillars has proven highly efficient. This approach allows not only to collect the starting material, but also to create additional conditions for the activation and reproduction of the entomophage directly in the field.

We used *E. kuehniella* and *G. mellonella* as laboratory hosts, a standard practice for mass breeding of *H. hebetor* [4,12]. However, it should be borne in mind that prolonged cultivation on alternative hosts can lead to a weakening or change in the parasitoid’s host range [34]. In this regard, we recommend periodically (at least 1–2 times a year) reintroducing field populations or reinforcing laboratory cultures using freshly harvested material from the field to preserve initial aggressiveness and specialization.

When interpreting the revealed differences between the two groups of *H. hebetor*, it is necessary to take into account the specifics of the formation of the initial laboratory cultures. Although individuals from different geographical points were maintained separately, within each point all adults that emerged from different cassettes and from different host species were combined into a single colony without maintaining separate maternal lines. This allows for the possibility of mixing genetically distinct lines within each point, as well as a founder effect. It should be taken into account that the colonies were maintained on different host species (*E. kuehniella* and *G. mellonella*), which could have influenced differentiation in host suitability. However, short-term rearing (2 generations) minimizes the likelihood of laboratory adaptation and directional selection, allowing the revealed differences to be considered predominantly as a reflection of initial field variability. To finally separate these effects, comparative studies of field and laboratory populations, as well as molecular-genetic analysis, are required. Thus, the revealed phenotypic differences between the groups should be considered preliminary, and their status as separate races requires confirmation by molecular-genetic methods and studies of reproductive isolation.

Based on the results obtained, we propose the following strategy for the use of *H. hebetor* in corn agrocenoses of the Krasnodar Territory: to carry out releases at an average daily temperature of 26–28 °C and humidity of 60–70% (optimal conditions for parasitoid activity); to use supporting cassettes with host caterpillars to stimulate reproduction and fixation of the parasitoid in the agrocenosis; and to combine the biological method with other elements of an integrated protection (agrotechnical techniques, pheromone monitoring, gentle insecticides, if necessary).

Despite the results, several open questions remain.

The genetic nature of the differences between the identified groups of *H. hebetor*. It is necessary to carry out a molecular genetic analysis to confirm the presence of genetic differentiation between the identified groups, and not just phenotypic specialization.

Prospects for the use of other populations. There may be populations with different host spectra (for example, specialized on leafrollers or other types of scoops) that require further monitoring.

## 5. Conclusions

1. As a result of the conducted study, the intraspecific differentiation of *H. hebetor* populations by host range was revealed. As a result of a laboratory assessment of two populations from the Krasnodar Territory, their differences in host suitability were established: the pyralid-leafroller population (Temryuksky district) effectively parasitizes the caterpillars of *O. nubilalis*, *E. zinckenella*, *T. viridana*, *C. pomonella*, and *G. funebrana*. Still, it does not infect the owlet moths like *H. armigera* and *P. candefacta*. The owlet-pyralid population (the central zone of the edge) has high paralyzing activity against all tested species; however, successful parasitization and development of offspring were observed only in *H. armigera* (87%), *O. nubilalis* (91%), and *E. zinckenella* (55.6%). In contrast, on leafroller moths, the parasitoid does not develop.

2. The dependence of the effectiveness of infection on the age structure of cotton bollworm caterpillars has been established. Laboratory experiments have shown that *H. hebetor* did not affect younger caterpillars (the proportion of paralyzed and parasitized specimens was 0%). In contrast, in middle-aged and older caterpillars, imago flight was 54.5% and 78.1%, respectively. This indicates the need for entomophage releases during periods when vulnerable age groups predominate in the pest population.

3. In comparative field observations under release conditions, with the pyralid–leafroller group released against the European corn borer at the beginning of tassel growth (rate: 1400 individuals/ha), damage was observed in 16% of corn plants in the treated plot compared with 40% in the control plot. With the owlet–pyralid group released against the cotton bollworm during flowering and early grain formation (two releases with a 5–7-day interval), damage was observed in 13% of corn ears in the treated plot compared with 65.0% in the control plot. Under release conditions, parasitization of cotton bollworm larvae reached 87.0%, with 88.3% adult emergence from the formed cocoons. Because these field comparisons used one treated and one control plot, with three sampling units per plot rather than independent plot-level replicates, these observations provide preliminary field evidence and should not be interpreted as experimentally demonstrated efficacy.

The obtained data confirm the presence of intraspecific differences in host suitability between the two studied groups of *H. hebetor*. However, it should be emphasized that the revealed differences are phenotypic in nature and require additional confirmation by molecular-genetic methods and studies of reproductive isolation. The obtained data should be considered preliminary, indicating the advisability of a differentiated approach to selecting a biocontrol agent population depending on the target pest.

Thus, preliminary testing of each natural population of *H. hebetor* for host range before its mass breeding and application helps to increase the effectiveness of the biological control. The revealed differences in the host spectrum confirm that there are different groups within the species, and using a “universal” population without assessing its host suitability can reduce the effectiveness of biological protection.

Captured and adapted to laboratory conditions, *H. hebetor* populations successfully reproduce on standard hosts (mill moth and greater wax moth), adapt well to field conditions, and demonstrate high biological potential to suppress key Lepidoptera pests of corn (European corn borer and cotton bollworm) as part of biological plant protection.

## Figures and Tables

**Figure 1:**
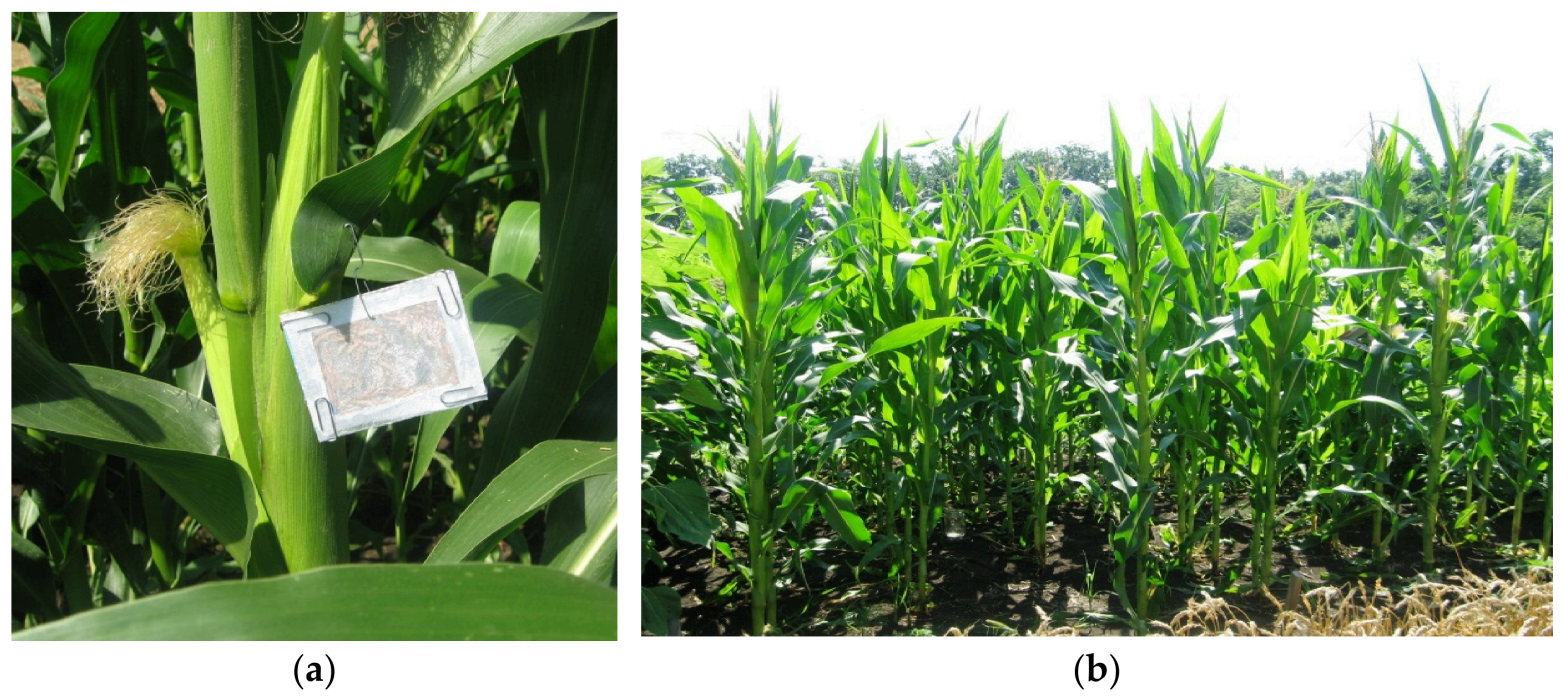
Cassettes with caterpillars for trapping *H. hebetor* (**a**) in the corn cenosis (**b**).

**Figure 2:**
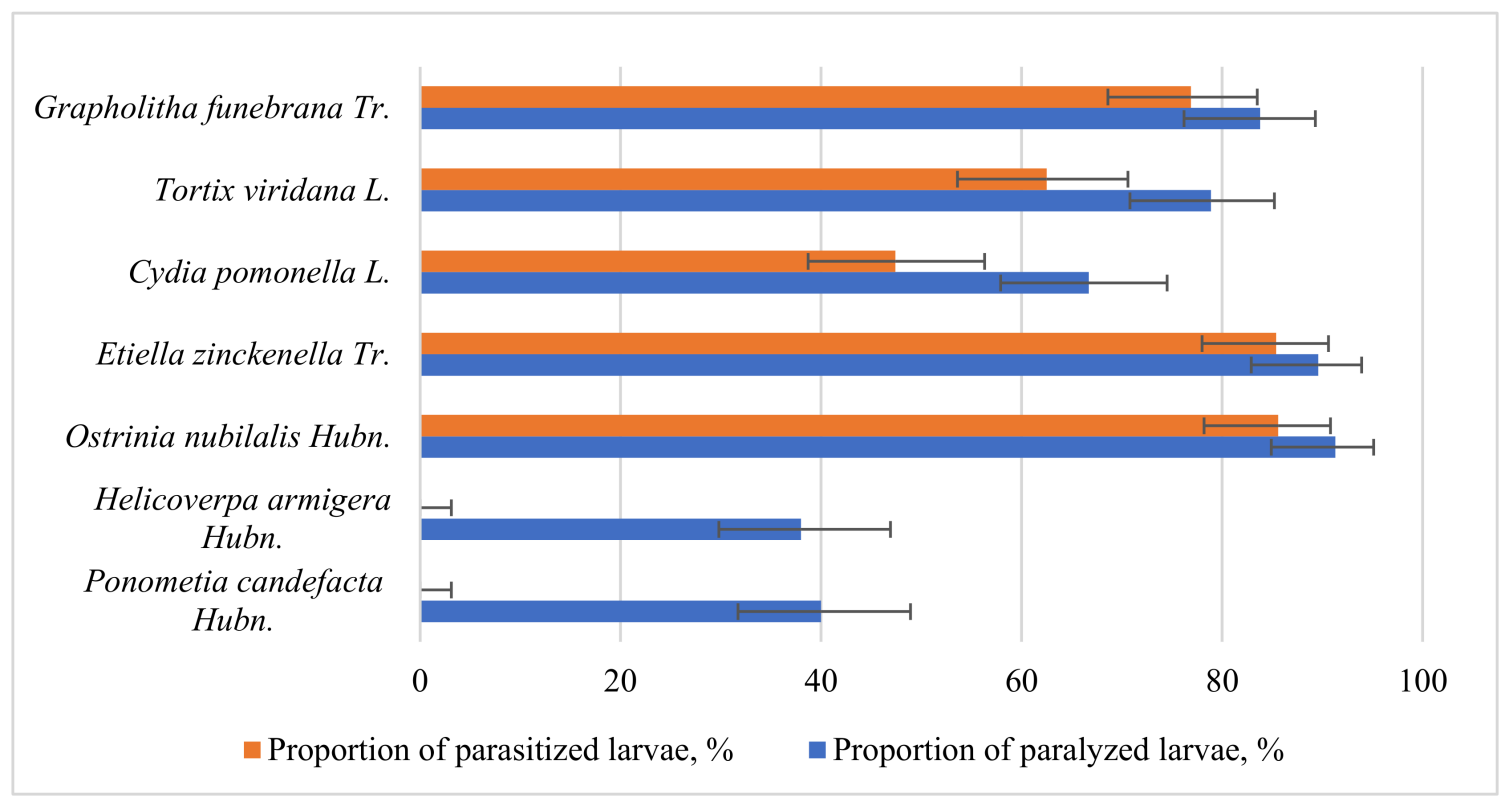
Host suitability and parasitic activity of the pyralid–leafroller group of *H. hebetor*. For each host species, 120 larvae were tested (*n* = 120). Data are presented as proportions of the total number of larvae offered. The experiment was conducted as a single pooled sample without independent replicates. Error bars represent 95% confidence intervals for proportions calculated using the Wilson method.

**Figure 3:**
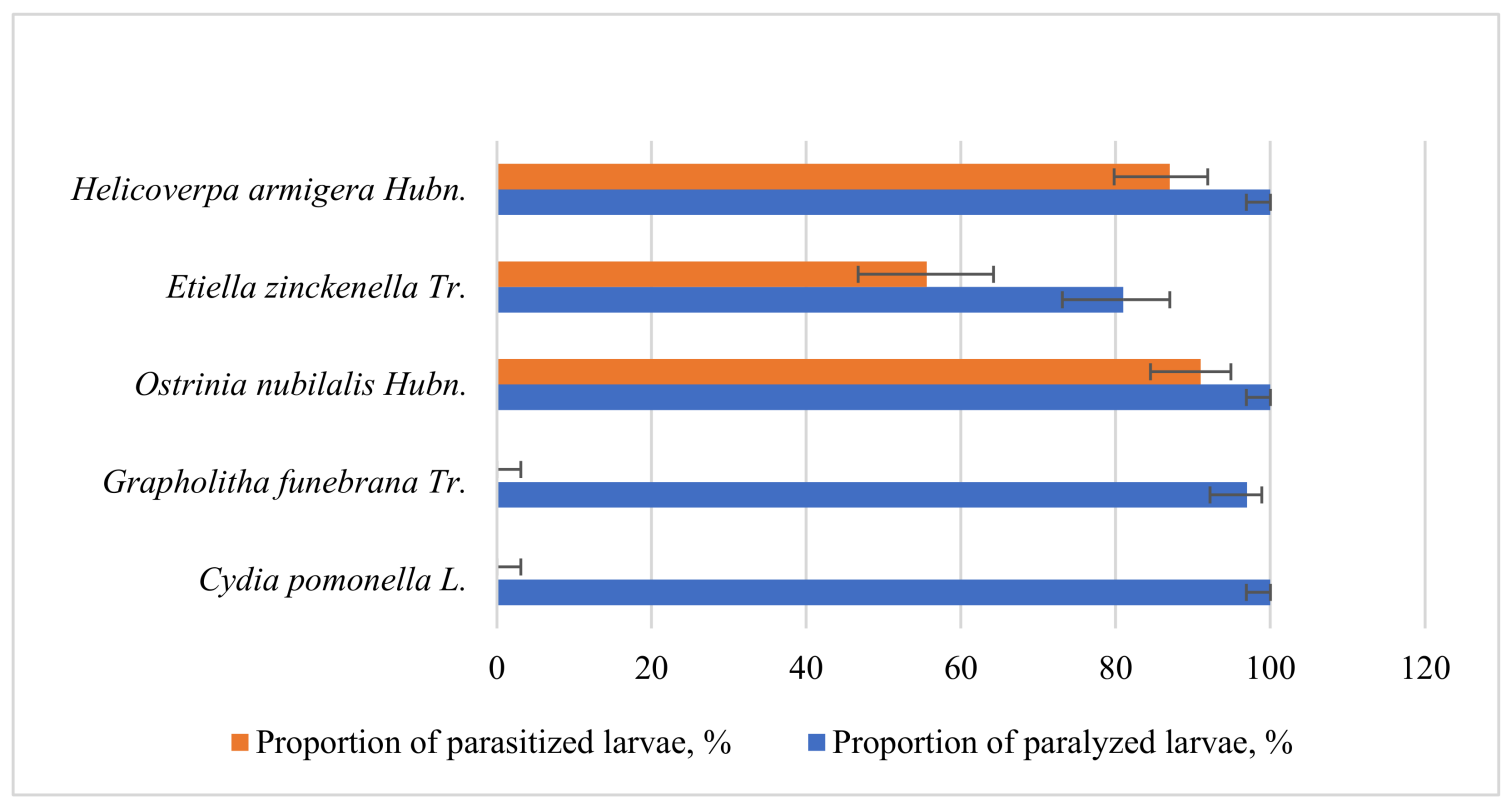
Host suitability and parasitic activity of the owlet–pyralid group of *H. hebetor*. For each host species, 120 larvae were tested (*n* = 120). Data are presented as proportions of the total number of larvae offered. The experiment was conducted as a single pooled sample without independent replicates. Error bars represent 95% confidence intervals for proportions calculated using the Wilson method.

**Figure 4:**
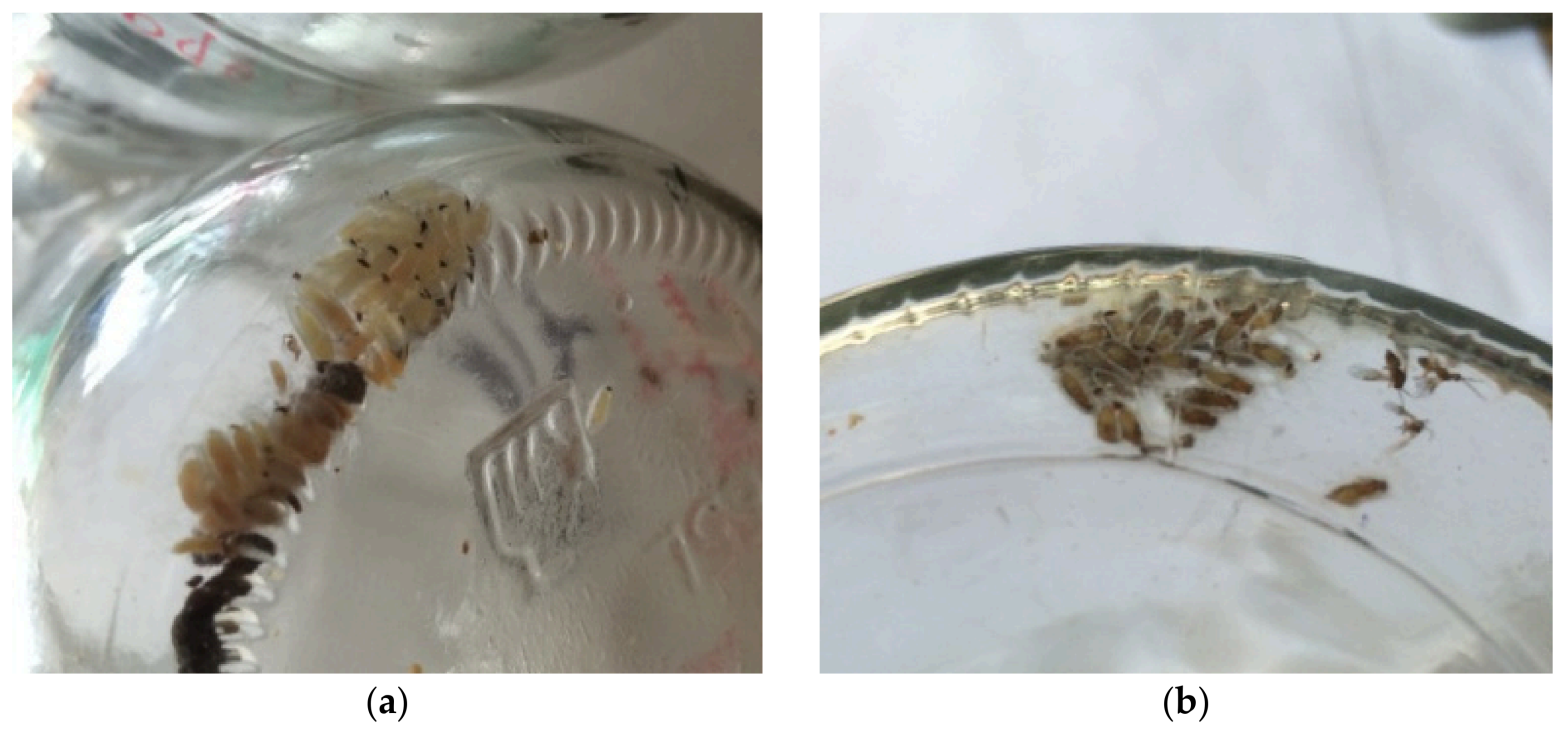
Parasitic activity of *H. hebetor* in relation to Pyralidae: cocoon formation on caterpillars of *O. nubilalis* (**a**); cocoon formation on caterpillars of *E. zinckenella* (**b**).

**Table 1:** Parasitic activity of ectoparasitoid *H. hebetor* in relation to the cotton bollworm *H. armigera*, laboratory conditions.

Cotton Bollworm, Caterpillars	Number of Caterpillars, Specimens	Of These:		Number of Cocoons Formed, Specimens	Number of Parasitoids That Have Flown out, %
		Paralyzed, %	Parasitized, %		
older age	42	100 ^d^ *	76.19 ^d^	32 ^d^	78.1 ^c^
middle-aged	34	100 ^c^	64.71 ^c^	22 ^c^	54.5 ^b^
younger age	37	0 ^a^	0 ^a^	0 ^a^	0 ^a^

* Note: there are no statistically significant differences between variants marked with the same letter indexes within the columns according to the Duncan criterion, with a 95% probability level.

**Table 2:** Comparative field observations of *H. hebetor* under release conditions against cotton bollworm on corn (M (mean) ± SD (standard deviation)).

Number of Caterpillars, Specimens	Of These, Proportion		Number of Parasitoid Cocoons Formed, Specimens	Proportion of Emerged Adults, %
	Paralyzed, %	Parasitized, %		
69.0 ± 8.7	100 ± 0.0	87.0 ± 7.2	234.3 ± 13.01	88.3 ± 0.5

## Data Availability

Dataset available on request from the authors.
